# A Low Excitation Working Frequency Capacitively Coupled Contactless Conductivity Detection (C^4^D) Sensor for Microfluidic Devices

**DOI:** 10.3390/s21196381

**Published:** 2021-09-24

**Authors:** Yuchen He, Qiang Huang, Yu He, Haifeng Ji, Tao Zhang, Baoliang Wang, Zhiyao Huang

**Affiliations:** State Key Laboratory of Industrial Control Technology, College of Control Science and Engineering, Zhejiang University, Hangzhou 310027, China; hyc19940615@zju.edu.cn (Y.H.); huang_q@zju.edu.cn (Q.H.); hyhelen@zju.edu.cn (Y.H.); zhtao@zju.edu.cn (T.Z.); wangbl@zju.edu.cn (B.W.); zy_huang@zju.edu.cn (Z.H.)

**Keywords:** contactless conductivity detection, capacitively coupled contactless conductivity detection (C^4^D), microfluidic, lab on a chip

## Abstract

In this work, a new capacitively coupled contactless conductivity detection (C^4^D) sensor for microfluidic devices is developed. By introducing an LC circuit, the working frequency of the new C^4^D sensor can be lowered by the adjustments of the inductor and the capacitance of the LC circuit. The limits of detection (LODs) of the new C^4^D sensor for conductivity/ion concentration measurement can be improved. Conductivity measurement experiments with KCl solutions were carried out in microfluidic devices (500 µm × 50 µm). The experimental results indicate that the developed C^4^D sensor can realize the conductivity measurement with low working frequency (less than 50 kHz). The LOD of the C^4^D sensor for conductivity measurement is estimated to be 2.2 µS/cm. Furthermore, to show the effectiveness of the new C^4^D sensor for the concentration measurement of other ions (solutions), SO_4_^2−^ and Li^+^ ion concentration measurement experiments were also carried out at a working frequency of 29.70 kHz. The experimental results show that at low concentrations, the input-output characteristics of the C^4^D sensor for SO_4_^2−^ and Li^+^ ion concentration measurement show good linearity with the LODs estimated to be 8.2 µM and 19.0 µM, respectively.

## 1. Introduction

Nowadays, due to their advantages in terms of portability and high efficiency, microfluidic devices receive increasing attention in many fields, such as biological and healthcare measurement [1,2,3,4,5], chemistry analysis [6,7,8,9,10], and pollution analysis [11]. Electrical conductivity is a basic and important property of electrolyte solution, biomaterial or fluid [12,13]. Different conductivity detection sensors have been studied in the research fields of microfluidic devices [14,15,16]. However, conventional conductivity detection techniques cannot meet the growing requirements of microfluidic devices [16,17,18]. The classic contact conductivity detection sensors have some disadvantages, such as the polarization effect and electrochemical erosion [12,13,16,17,18], as a result of their electrodes being in direct contact with the measured fluid [16,17,18]. Furthermore, it is difficult to fix a contact conductivity sensor into such a small channel [16,17,18,19,20,21,22].

Capacitively coupled contactless conductivity detection (C^4^D), is an approach for realizing the online conductivity measurement [16,17,18]. Because the electrodes of the C^4^D sensors are not in contact with the measured fluid, the drawbacks of the polarization effect and electrochemical erosion can be avoided [16,17,18]. Due to its advantages, the C^4^D method has received great attention since it appeared. It has been used for organic ion detection and inorganic ion detection by measuring the conductivity of the solution (the conductivity is determined by the concentration of ions in solution) and, hence, has been used to realize food analysis, pharmaceutical analysis, clinical analysis, environment analysis and the analysis of beta-lactam antibiotics, lidocaine, fentanyl, diphenhydramine and polyamines [16,17,18].

However, although C^4^D has great potential and the prospect of broad applications, it is still a developing technique [16,17,18]. With the decrease of the scale of microfluidic devices, the cross-section area of the microchannel will become very small. The equivalent resistance of a solution with low conductivity in such a small microchannel will become very significant [19,20]. As conventional C^4^D sensors have difficulty in detecting solution with such large equivalent resistance, the measurement performance for low conductivity solution in microchannels should be improved [19,20]. However, few research works which use the C^4^D sensor to investigate solution with conductivity of less than 0.2 mS/cm [16,17,18,19,20,21,22] have been reported. As conductivity is proportional to the ion concentration of the solution (low concentration solution), a C^4^D sensor with an LOD greater than 0.2 mS/cm cannot meet the requirements of many applications concerning ion detection at low concentrations [16,17,18]. For example, ion chromatograph (IC) is an important method for ion separation and detection, and has been used in the field of microfluidic devices [6,7]. In IC systems, conductivity detectors are the most common detectors [6,7,19,20]. Because the background conductivity of eluents in IC systems can result in great trouble for the conductivity detectors in detecting the concentration of ions, IC systems usually use low conductivity eluents (non-suppressed IC) or use suppressors to greatly reduce the background conductivity of eluents (suppressed IC) before detection [6,7,19,20]. Nowadays, suppressed IC has reached a background conductivity of less than 3 µS/cm before detection, which is much lower than 0.2 mS/cm [19,20]. Therefore, to better bring the potential power of C^4^D and extend the application fields of C^4^D, and it is necessary to further improve the measurement performance of the C^4^D sensor, especially the LOD of the C^4^D sensor.

**Figure 1:**
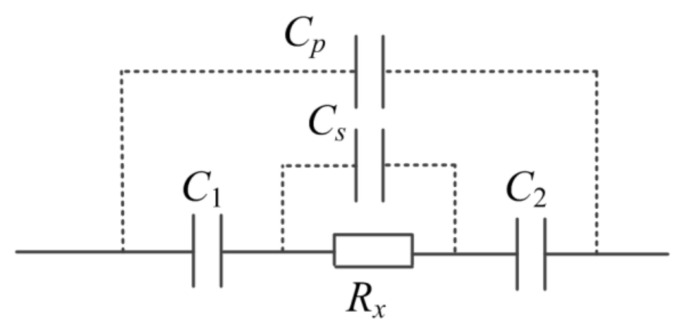
The equivalent circuit of a typical C^4^D sensor.

The intrinsic property of the electrolyte solution and the particularity of microfluidic devices are the crux. Figure 1 illustrates the equivalent circuit of a typical C^4^D sensor, where $C_{1}$ and $C_{2}$ are the coupling capacitances formed by the two electrodes of the C^4^D sensor, the insulating channel wall, and the measured fluid in the channel. According to the classical theory of electrolyte solution, the electrolyte solution could be equivalent to a parallel connection of a resistor $R_{x}$ and a capacitance $C_{s}$ [12,13]. Besides, there exists a stray capacitance $C_{p}$ which arises from direct coupling between the two electrodes of the C^4^D sensor.

With the decrease of the scale of the microfluidic device, the value of $R_{x}$ will significantly increase and become comparable to that of the impedance of $C_{s}$. The influence of $C_{s}$ on the conductivity measurement cannot be neglected. Furthermore, due to the scale, it is difficult to introduce shielding into the microfluidic device to overcome the influence of the stray capacitance $C_{p}$. Thus, from the viewpoint of impedance measurement, the impedances produced by $C_{s}$ and $C_{p}$ are background signals, which will limit the measurement performance of the C^4^D sensor.

$C_{s}$ and $C_{p}$ are in parallel with the solution resistor $R_{x}$. Their influences on the conductivity measurement could be effectively neglected only if the impedance of $C_{s}$ and the impedance of $C_{p}$ are much greater than $R_{x}$, i.e., $\frac{1}{j2\pi fC_{s}}\gg R_{x}$ and $\frac{1}{j2\pi fC_{p}}\gg R_{x}$. As we know, the value of $R_{x}$ in microfluidic devices is very large. An effective way to overcome the influences of $C_{s}$ and $C_{p}$ on the measurement is to reduce the working frequency f. However, as shown in Figure 1, with the decrease of the working frequency f, the background impedances produced by $C_{1}$ and $C_{2}$ $\left(\frac{1}{j2\pi fC_{1}}+\frac{1}{j2\pi fC_{2}}\right)$ will increase. Such results are not beneficial for the $R_{x}$ measurement, because $C_{1}$ and $C_{2}$ are in series with $R_{x}$. Thus, in microfluidic devices, the measurement of $R_{x}$ is in dilemmatic situation. This fact causes difficulty in lowering the working frequency of the C^4^D sensor and difficulty in improving the LOD of the conventional C^4^D sensor (the working frequency of conventional C^4^D sensor is usually in the 100 s of kHz or even in the MHz range) [16,17,18,19,20,21,22,23,24,25,26,27,28,29,30,31,32,33,34].

Currently, Huck et al. [21] and Zhang et al. [19,20] have made efforts to solve this problem. Huck et al. used a high dielectric constant material (barium strontium titanate) as the channel wall between the electrodes and the channel. With this material, the coupling capacitances are much greater and the background impedance is much smaller, and the working frequency is less than 50 kHz [21]. Zhang et al. optimized the channel size and the electrode length, introduced a shielding and reached a working frequency of less than 50 kHz [19,20]. Huck et al. and Zhang et al. have achieved great progress, which makes it possible for C^4^D sensors to approach a working frequency of less than 50 kHz effectively, and provides useful references for other researchers. However, in the field of microfluidic devices, the dielectric constants of materials (e.g., glass, PMMA and PDMS) are usually not high [16,17,18,24,25,26,27,28,29,30,31,32,33,34]. It is difficult to introduce shielding into microfluidic devices and the optimization of electrodes and the channel size will more or less introduce some limitations in practical applications [24,25,26,27,28,29,30,31,32,33,34]. Meanwhile, recently, few research works which use C^4^D sensors at a low working frequency and investigate conductivity of less than 0.2 mS/cm have been reported [16,17,18,19,20]. More research should be undertaken in order to propose a more effective C^4^D sensor.

In this work, a new C^4^D sensor is developed for microfluidic devices with the introduction of an LC circuit. By the adjustment of the value of the inductor and the capacitance in the LC circuit, the C^4^D sensor can work at a low working frequency (less than 50 kHz) and the LODs of the C^4^D sensor for conductivity measurement and ion concentration measurement will be improved. A microfluidic device (microfluidic chip) with the channel of 500 µm × 50 µm and a new C^4^D sensor is developed. Conductivity measurement experiments are carried out with KCl solutions to verify the effectiveness of the new C^4^D sensor and to identify out its input-output characteristics. The SO_4_^2^^−^ ion and Li^+^ ion concentration measurement is then carried out to investigate the performance of the developed sensor for concentration measurement with other ions (solutions).

## 2. Materials and Methods

### 2.1. Material and Reagents

A SU-8 3025 was purchased from Microchem, Westborough, MA, USA. The photomask was designed using CorelDRAW software and printed in Luolanxin Laser Technology (Shenzhen, China). PDMS prepolymers A and B were purchased from Sylgard 184, Dow Corning, Midland, MI, USA. Trimethylchlorosilane was purchased from Sigma-Aldrich, St Louis, MO, USA. Potassium chloride (KCl) was purchased from Sigma-Aldrich, St Louis, MO, USA. Potassium sulfate (K_2_SO_4_) and lithium chloride (LiCl) were purchased from Aladdin-Holdings Group, Beijing, China. KCl solution was obtained by dissolving KCl in deionized water. Solution of SO_4_^2^^−^ ion and Li^+^ ion was prepared by dissolving K_2_SO_4_ and LiCl in deionized water. The printed circuit boards (PCB) with electrodes were made to order by the electronics workshop and were processed according to standard instructions.

### 2.2. Microfluidic Device Fabrication

**Figure 2:**
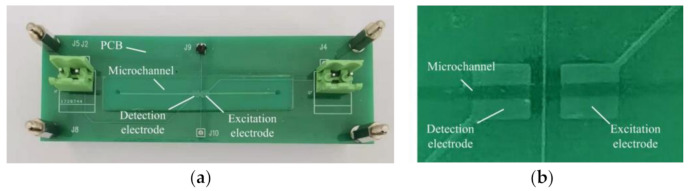
(**a**) The construction of the microfluidic device. (**b**) The photograph of electrodes and microchannel in the microfluidic device.

The microfluidic device was a microfluidic chip bonded on a PCB with electrodes of the C^4^D sensor. The microfluidic chip was fabricated via standard photolithography. The SU-8 3025 mold with a thickness of 50 µm was fabricated on a clean silicon wafer and was treated with trimethylchlorosilane to facilitate the stripping of the polydimethylsiloxane from the mold. The width and the length of channel in the mold is 500 µm and 50 mm. A portion (20 g) of PDMS prepolymers A and B with a ratio of 10:1 was poured on the mold. After baking at 85 °C for 90 min, a PDMS block with the channel was formed. The PDMS block with the channel was then carefully peeled off from the mold and two 1.5 mm holes were punched as the inlet and the outlet of the microfluidic chip. The channel side of the PDMS block was then bonded to a pre-prepared PDMS film with a thickness of 20 µm via oxygen plasma treatment to form the microfluidic chip. Finally, the microfluidic chip and a pre-prepared PCB with two electrodes of C^4^D sensors (the widths of the two electrodes were 2 mm and the gap between the electrodes was 1 mm) were both plasma-activated and bonded together to form the microfluidic device, as shown in Figure 2a,b. Thus, between the channel and the electrodes, there is only a PDMS film with a thickness of 20 µm.

### 2.3. The Development of the New C^4^D Sensor

**Figure 3:**
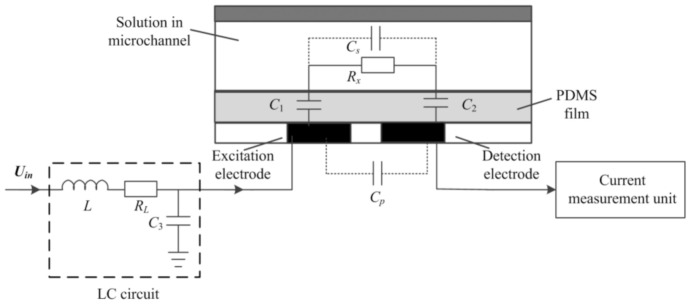
The construction of the new C^4^D sensor.

**Figure 4:**
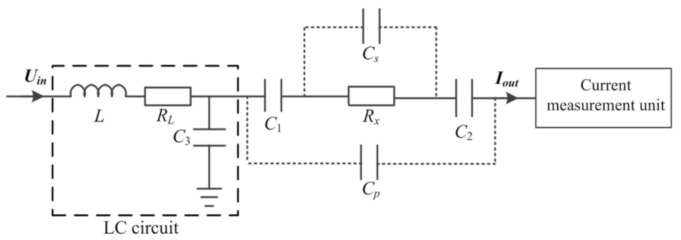
The equivalent circuit of the new C^4^D sensor.

This new C^4^D sensor tends to implement conductivity measurements at a low working frequency to overcome the influence of $C_{s}$, and $C_{p}$ on the measurement while eliminating the background impedance of the coupling capacitance by introducing an LC circuit to the detection path. Figure 3 shows the construction of the new C^4^D sensor. Figure 4 shows the equivalent circuit of the new C^4^D sensor. The AC source is connected to the introduced inductance module L (its internal resistor is $R_{L}$). The introduced capacitance $C_{3}$ is grounded. The detection path is connected with the middle point of L and $C_{3}$. When an AC voltage $U_{in}\;$ is applied, a current $I_{out}$ will flow through the detection path. Thus, the contactless conductivity detection is realized by measuring $I_{out}$.

As mentioned in Section 1, the new C^4^D sensor should be working at a low working frequency and the influences of $C_{s}$ and $C_{p}$ should be neglected. Thus, assuming $\frac{1}{j2\pi fC_{s}}\gg R_{x}$ and $\frac{1}{j2\pi fC_{p}}\gg R_{x}$, from Figure 3, the output current $I_{out}$ is:

(1) $$I_{out}=\frac{U_{in}}{\left(1-4\pi^{2}f^{2}LC_{3}\right)R_{x}+\frac{C_{3}+C_{e}}{C_{e}}R_{L}-j\left(\frac{1}{2\pi fC_{e}}-2\pi fL\frac{C_{e}+C_{3}}{C_{e}}-2\pi fR_{x}R_{L}C_{3}\right)}$$

where $C_{e}=C_{1}C_{2}/\left(C_{1}+C_{2}\right)$ and hence $-j\frac{1}{2\pi fC_{e}}$ is the background impedance of the coupling capacitance.

Let $-j\left(\frac{1}{2\pi fC_{e}}-2\pi fL\frac{C_{e}+C_{3}}{C_{e}}-2\pi fR_{x}R_{L}C_{3}\right)=0$. The working frequency f is determined by the following equation (at this working frequency, $-j\left(\frac{1}{2\pi fC_{e}}-2\pi fL\frac{C_{e}+C_{3}}{C_{e}}-2\pi fR_{x}R_{L}C_{3}\right)=0$ and so the background impedance of the coupling capacitance is eliminated):

(2) $$f=\frac{1}{2\pi }\sqrt{\frac{1}{L\left(C_{e}+C_{3}\right)+R_{x}R_{L}C_{e}C_{3}}}$$

Furthermore, to determine the working frequency f conveniently, the $R_{x}R_{L}C_{e}C_{3}$ is much less than $L\left(C_{e}+C_{3}\right)$, i.e., $R_{x}R_{L}C_{e}C_{3}\ll L\left(C_{e}+C_{3}\right)$. Thus, the working frequency is mainly determined as:

(3) $$f=\frac{1}{2\pi }\sqrt{\frac{1}{L\left(C_{e}+C_{3}\right)}}$$

For most conditions of conductivity measurement in microfluidic devices, the assumption $R_{x}R_{L}C_{e}C_{3}\ll L\left(C_{e}+C_{3}\right)$ can be satisfied. For example, in this work, to satisfy $\frac{1}{j2\pi fC_{s}}\gg R_{x}$ and $\frac{1}{j2\pi fC_{p}}\gg R_{x}$, the working frequency is set to be less than 50 kHz by adjusting the values of L and $C_{3}$ ($C_{s}$ is less than 20 fF and $C_{p}$ is approximately 100 fF). This working frequency can be realized when the value of L and $C_{3}$ is 700.0 mH and 14 pF according to Equation (2) ($C_{e}$ is approximately 0.6 pF). At the conductivity range of 10 µS/cm–100 mS/cm, the maximum value of $R_{x}$ is approximately 50.0 MΩ (at the conductivity of 10 µS/cm). Thus, the maximum value of $R_{x}R_{L}C_{e}C_{3}$ is approximately $9.2\times 10^{-13}$ Ω^2^F^2^, while $L\left(C_{e}+C_{3}\right)$ is approximately $1.0\times 10^{-11}$ Ω^2^F^2^. Thus, the assumption $R_{x}R_{L}C_{e}C_{3}\ll L\left(C_{e}+C_{3}\right)$ is satisfied when the conductivity is higher than 10 µS/cm.

Thus, the output of the detection path $I_{out}$ is:

(4) $$I_{out}=\frac{1}{\left(1-4\pi^{2}f^{2}LC_{3}\right)R_{x}+\frac{C_{3}+C_{e}}{C_{e}}R_{L}}U_{in}=\frac{1}{k_{1}R_{x}+\frac{1}{k_{1}}R_{L}}U_{in}$$

where $k_{1}=C_{e}/\left(C_{3}+C_{e}\right)$.

Meanwhile, because it is in an electrolyte solution, $R_{x}$ is inversely proportional to the conductivity σ of the electrolyte solution [12,13], i.e.,$\;R_{x}=k_{2}/\sigma$, where $k_{2}$ is a coefficient. Thus, $I_{out}$ could be rewritten as:

(5) $$I_{out}=\frac{1}{k_{1}k_{2}\frac{1}{\sigma }+\frac{1}{k_{1}}R_{L}}U_{in}=\frac{k_{1}\sigma }{k_{1}{}^{2}k_{2}+R_{L}\sigma }U_{in}$$

Because L,$\;C_{e}$, $C_{3}$, $R_{L}$ and f are all known parameters, $k_{1}$ and $k_{2}$ could be pre-determined. Thus, the measurement of conductivity σ can be realized by the measurement of $I_{out}$.

The influence of solution capacitance and stray capacitance is overcome, while the background impedance of the coupling capacitance is also eliminated and the dilemma that is caused by solution capacitance, stray capacitance and coupling capacitance is solved. The working frequency can be determined by Equation (3) conveniently.

**Figure 5:**
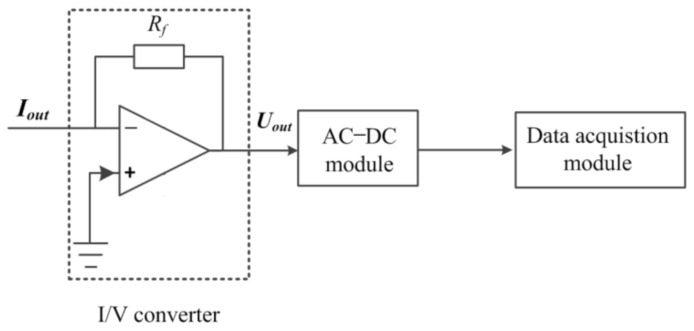
The flowchart of the current measurement.

In this work, the measurement of $I_{out}$ is realized by a current measurement unit. Figure 5 shows the flowchart of the current measurement (the construction of the current measurement unit). It consists of an I/V converter, an AC-DC module and a data acquisition module.

After the operation of the I/V converter, $I_{out}$ is controverted to $U_{out}$:

(6) $$U_{out}=-R_{f}I_{out}$$

$U_{out}$ is operated by the AC-DC module and then sampled by the data acquisition module.

### 2.4. The Experimental Setup

**Figure 6:**
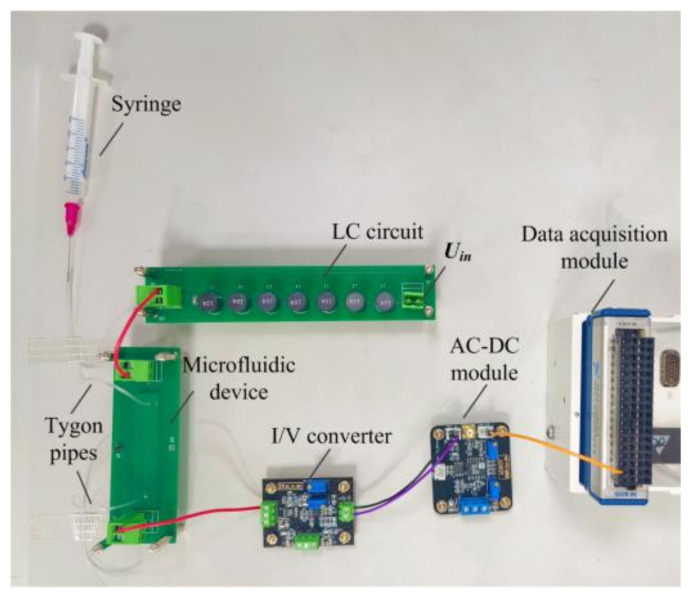
The photo of the experimental setup.

Figure 6 shows the photo of the experimental setup. In this work, fixed tygon pipes serve as the inlet and outlet of the microfluidic devices. A syringe is used to inject the solution into the channel of the microfluidic devices through the tygon pipes. The A_1_ in the I/V converter is AD825 and *R*_f_ is 3.3 MΩ. The AC-DC module is purchased from Kangwei, Chengdu, China. The data acquisition module is NI cDAQ-9172.

## 3. Results and Discussion

### 3.1. Conductivity Measurement Experiments

To verify the effectiveness of the new C^4^D sensor, conductivity measurement experiments were carried out with the KCl solution. A KCl solution, with the conductivity ranging from 1 µS/cm to 100 mS/cm, was injected into the microchannel of the microfluidic devices. A signal generator (CA1640-02, RIGOL Technologies Inc., Beijing, China) was used as the AC source (sinusoidal waves) and the voltage was 0.25 Vpp. The measurement performances of the C^4^D sensor under different working frequencies (29.09 and 47.57 kHz) were investigated. For the working frequency of 29.09 kHz, the corresponding values of L and $C_{3}$ were 700 mH and 42 pF, respectively. For the working frequency of 47.57 kHz, the corresponding values of L and $C_{3}$ were 700 mH and 14 pF, respectively.

**Figure 7:**
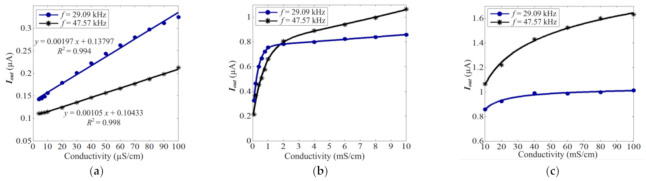
The input-output characteristics of the new C^4^D sensor for conductivity measurement with the working frequency of 29.09 and 47.57 kHz. (**a**) The detailed input-output characteristics under the conductivity range of 1–100 µS/cm. (**b**) The detailed input-output characteristics under the conductivity range of 100 µS/cm–10 mS/cm. (**c**) The detailed input-output characteristics under the conductivity range of 10–100 mS/cm.

Figure 7 shows the input-output characteristics (1 µS/cm–100 mS/cm) of the new C^4^D sensor for conductivity measurements with the working frequency of 29.09 and 47.57 kHz, respectively.

The experimental results indicate that the new C^4^D sensor is effective. It can successfully realize conductivity measurements with a low working frequency (less than 50 kHz). The input-output curve has sufficient monotonousness and satisfactory sensitivities. When the conductivity is less than 100 µS/cm, the input-output characteristics of the C^4^D sensor for conductivity measurements show good linearity. Meanwhile, with the low working frequency, when the conductivity is less than 10 µS/cm, the C^4^D sensors still have considerable sensitivities, as shown in Figure 7a. The LOD of the C^4^D sensor for the conductivity measurement is estimated to be 2.2 µS/cm at a signal-to-noise ratio of 3.

Meanwhile, the experimental results indicate that in a lower conductivity range, as shown in Figure 7a,b, lower working frequencies have higher sensitivity. This means that a lower working frequency is of benefit to the measurement of the lower solution conductivity. In a higher conductivity range (as shown in Figure 7b,c), a higher working frequency has a higher sensitivity; with the increase of the working frequency, the slope of the input-output characteristics curve increases. This means that a higher working frequency is of benefit to the measurement of the higher solution conductivity.

**Figure 8:**
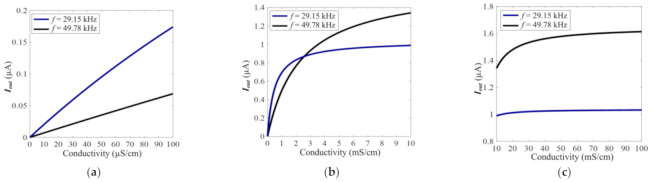
The theoretical curves of Equation (5) which show the complex relationship between $I_{out}$ and σ. (**a**) The detailed theoretical curves of Equation (5) under the conductivity range of 1–100 µS/cm. (**b**) The detailed theoretical curves of Equation (5) under the conductivity range of 100 µS/cm–10 mS/cm. (**c**) The detailed theoretical curves of Equation (5) under the conductivity range of 10–100 mS/cm. Notations: The values of L and $C_{3}$ are set as the same as the practical C^4^D sensor. $R_{L}$ is set as 2200 and 1200 Ω, respectively. $C_{e}$ is set as 0.6 pF.

This phenomenon could be explained by Equation (5). Figure 8 shows the theoretical curves of Equation (5) which shows the complex relationship between $I_{out}$ and σ. Comparing Figure 7; Figure 8, it can be seen that the experimental results are in accord with the theoretical analysis. Equation (5) is a complex and monotonous function in essence.

**Figure 9:**
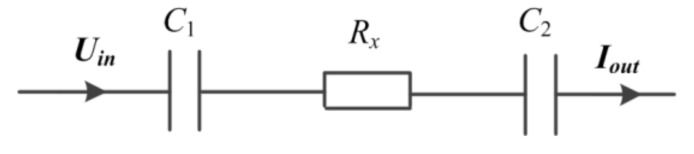
The equivalent of C^4^D sensor without LC circuit (the equivalent circuit of conventional C^4^D sensor).

To better show the improvement that occurs with the introduction of the LC circuit, the difference of C^4^D sensor with and without the LC circuit is also discussed. Figure 9 shows the equivalent circuit without the LC circuit (the conventional C^4^D sensor). With a conventional C^4^D sensor, the relationship between $U_{in}$ and ***I_out_*** is (assuming the output is grounded):

(7) $$I_{out}=\frac{U_{in}}{R_{x}-j\frac{1}{2\pi fC_{1}}-j\frac{1}{2\pi fC_{2}}}=\frac{U_{in}}{\frac{k_{2}}{\sigma }-j\frac{1}{2\pi fC_{e}}}$$

**Figure 10:**
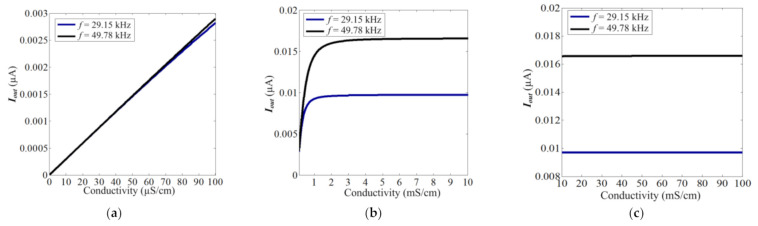
The theoretical curves of Equation (7) (the C^4^D sensor without the LC circuit). (**a**) The detailed theoretical curves of Equation (7) under the conductivity range of 1–100 µS/cm. (**b**) The detailed theoretical curves of Equation (7) under the conductivity range of 100 µS/cm–10 mS/cm. (**c**) The detailed theoretical curves of Equation (7) under the conductivity range of 10–100 mS/cm.

The theoretical curves of Equation (7) are shown in Figure 10. The working frequency is set as 29.15 and 49.78 kHz, and the excitation voltage is also set as 0.25 Vpp (the same as practical experiment and theoretical curves of C^4^D sensor with LC circuit).

Comparing Figure 8a,c and Figure 10a,c, it can be clearly seen that the output current and sensitivity of the C^4^D sensor with the LC circuit are approximately two orders of magnitude greater than that of the C^4^D sensor without the LC circuit (at the same working frequency and excitation amplitude). This is beneficial for the improvement of the LOD. Meanwhile, it can be found that at a conductivity range that is lower than 30 mS/cm, the C^4^D sensor with the LC circuit can show high sensitivity, while the C^4^D sensor without the LC circuit only shows high sensitivity at a conductivity of less than 2 mS/cm.

From the above discussion, it can be seen that the introduction of the LC circuit can effectively improve the measurement performance of the C^4^D sensor.

### 3.2. SO_4_^2−^ Ion and Li^+^ Ion Concentration Measurement Experiments

**Figure 11:**
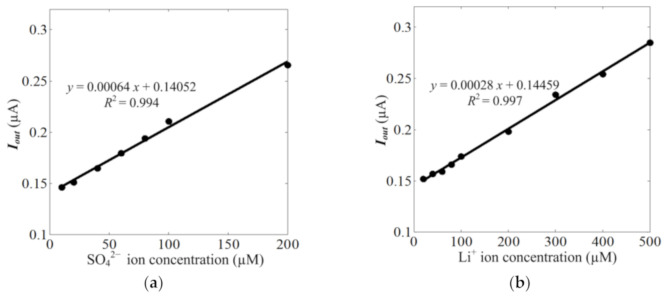
The experimental results of SO_4_^2−^ ion and Li^+^ ion concentration measurement. (**a**) The input-output characteristics of C^4^D sensor for SO_4_^2−^ ion concentration measurement. (**b**) The input-output characteristics of C^4^D sensor for Li^+^ ion concentration measurement.

To further verify the effectiveness of the new C^4^D sensor for ion concentration measurement, ion concentration measurement experiments were also carried out. Because SO_4_^2−^ ion is a typical ion that is analyzed in pollution analysis and Li^+^ ion can be recovered from sea water and used in many applications, SO_4_^2−^ ion and Li^+^ ion were selected and their concentration measurement experiments were also carried out. Because a low working frequency is beneficial for the LOD of the C^4^D sensor for conductivity measurement, the working frequency is set as 29.70 kHz (at a low concentration, the concentrations of ions are proportional to the conductivity of solution). A solution with a SO_4_^2−^ ion concentration that was less than 200 µM and a solution with a Li^+^ ion concentration that was less than 500 µM were injected into the microchannel of the microfluidic device (at this concentration range, the theoretical conductivity of the solution is in the linear range of the C^4^D sensor according to Section 3.1). Figure 11 shows the experimental results for the SO_4_^2−^ ion and Li^+^ ion concentration measurements.

From Figure 11, it can be found that the new C^4^D sensor is effective for SO_4_^2−^ ion and Li^+^ ion concentration measurements. The input-output characteristics of the C^4^D sensor for SO_4_^2−^ ion and Li^+^ ion concentration measurements show good linearity at a low concentration. The LODs of the C^4^D sensor for SO_4_^2−^ ion and Li^+^ ion concentration measurement are estimated to be 8.2 and 19.0 µM at a signal-to-noise ratio of three, respectively. These measurement results are comparable to that of chip-based ion chromatography with a contact conductivity detection sensor [6].

### 3.3. Discussion

From above analysis, it can be found that, with the introduction of the LC circuit, the unfavorable influence of solution capacitance and the stray capacitance can be overcome, while the background impedance of coupling capacitance can also be eliminated. Therefore, the dilemma caused by solution capacitance, stray capacitance and coupling capacitance is solved. The LODs of the C^4^D sensor for conductivity measurements and ion concentration measurements can be improved.

The new C^4^D sensor can successfully realize the conductivity measurement at a working frequency of less than 50 kHz (the working frequency for most conventional C^4^D sensors is 100 s of kHz or even in the MHz range). The LOD of the C^4^D sensor for conductivity measurements is improved and is estimated to be 2.2 µS/cm (few research works, which use the C^4^D sensor to investigate solution with conductivity less than 0.2 mS/cm, have been reported).

The research results also show that in a lower conductivity range (1–100 µS/cm), a lower working frequency is of benefit to the improvement of the LOD, while in a higher conductivity range (1–100 mS/cm), a higher working frequency is of benefit to the measurement of the higher solution conductivity.

In addition, the LODs of the C^4^D sensor for concentration measurements of SO_4_^2−^ ion and Li^+^ ion are 8.2 and 19.0 µM, respectively, which is comparable to that of chip-based ion chromatography with contact conductivity detection sensors.

Meanwhile, the measurement performance can be further improved with a decrease in the inductor’s internal resistance *R_L_*. In this work, we introduced an inductor with large inductance value. Because the introduced inductor is made of coils, large inductance leads to greater internal resistance $R_{L}$ (the internal resistance of the introduced inductor is usually greater than 1 kΩ). From Equations (1)–(5), it could be clearly found that the existence of the internal resistance $R_{L}$ causes much trouble for conductivity measurement and introduces an unfavorable influence on the measurement of the C^4^D sensor. This is the drawback of the new C^4^D sensor. To seek a more effective approach to develop an inductor with small internal resistance (such as implementing simulated inductor technique [35,36]) and, hence, to further improve the measurement performance of the C^4^D sensor will be our further research work.

## 4. Conclusions

In this work, a new C^4^D sensor for microfluidic devices, which can work at a low working frequency and has better LODs for conductivity measurement and ion concentration measurement, is developed.

In the new C^4^D sensor, with the introduction of an LC circuit, the working frequency can be lowered by the adjustment of the inductor L and the capacitance $C_{3}$ in the LC circuit. Accordingly, the C^4^D sensor can realize conductivity and ion concentration measurements at a low working frequency by adopting the proper value of L and $C_{3}$.

Compared with how conventional C^4^D sensors work under lower working frequencies, the sensitivity and the output current are here increased and thereby the LOD can also be improved.

Finally, it is necessary to indicate that the new C^4^D sensor has the potential to be further improved concerning its measurement performance with an inductor of small internal resistance. This can be realized with the use of simulated inductor module.

The new C^4^D sensor can be used for environmental analysis (such as the analysis of metal ions), food analysis (such as the analysis of amino acids), pharmaceutical and clinical analysis (such as the analysis of glucose and lactate) and has the prospect of broad applications.

## Data Availability

The data presented in this study are available on request from the corresponding author. The data are not publicly available due to protection of intellectual property.
